# Transcriptome Analysis Reveals Signature of Adaptation to Landscape Fragmentation

**DOI:** 10.1371/journal.pone.0101467

**Published:** 2014-07-02

**Authors:** Panu Somervuo, Jouni Kvist, Suvi Ikonen, Petri Auvinen, Lars Paulin, Patrik Koskinen, Liisa Holm, Minna Taipale, Anne Duplouy, Annukka Ruokolainen, Suvi Saarnio, Jukka Sirén, Jukka Kohonen, Jukka Corander, Mikko J. Frilander, Virpi Ahola, Ilkka Hanski

**Affiliations:** 1 Department of Biosciences, University of Helsinki, Helsinki, Finland; 2 Institute of Biotechnology, Genome Biology Program, University of Helsinki, Helsinki, Finland; 3 Lammi Biological Station, University of Helsinki, Lammi, Finland; 4 Science for Life Laboratory, Department of Biosciences and Nutrition, Karolinska Institutet, Stockholm, Sweden and Genome-Scale Biology Research Program & Institute of Biomedicine, University of Helsinki, Helsinki, Finland; 5 Department of Mathematics and Statistics, University of Helsinki, Helsinki, Finland; University of Basel, Switzerland

## Abstract

We characterize allelic and gene expression variation between populations of the Glanville fritillary butterfly (*Melitaea cinxia*) from two fragmented and two continuous landscapes in northern Europe. The populations exhibit significant differences in their life history traits, e.g. butterflies from fragmented landscapes have higher flight metabolic rate and dispersal rate in the field, and higher larval growth rate, than butterflies from continuous landscapes. In fragmented landscapes, local populations are small and have a high risk of local extinction, and hence the long-term persistence at the landscape level is based on frequent re-colonization of vacant habitat patches, which is predicted to select for increased dispersal rate. Using RNA-seq data and a common garden experiment, we found that a large number of genes (1,841) were differentially expressed between the landscape types. Hexamerin genes, the expression of which has previously been shown to have high heritability and which correlate strongly with larval development time in the Glanville fritillary, had higher expression in fragmented than continuous landscapes. Genes that were more highly expressed in butterflies from newly-established than old local populations within a fragmented landscape were also more highly expressed, at the landscape level, in fragmented than continuous landscapes. This result suggests that recurrent extinctions and re-colonizations in fragmented landscapes select a for specific expression profile. Genes that were significantly up-regulated following an experimental flight treatment had higher basal expression in fragmented landscapes, indicating that these butterflies are genetically primed for frequent flight. Active flight causes oxidative stress, but butterflies from fragmented landscapes were more tolerant of hypoxia. We conclude that differences in gene expression between the landscape types reflect genomic adaptations to landscape fragmentation.

## Introduction

Human land use has converted more than half of the area of many ecosystems and biomes on earth [1], and much of the remaining habitat has become severely fragmented [2], [3] to the detriment of innumerable species and populations [4],[5]. The long-term persistence of fragmented populations, called metapopulations [6], hinges on the processes of dispersal, colonization, founder events and gene flow in addition to demographic and evolutionary dynamics (local adaptation) [6],[7]. Apart from the direct demographic consequences of habitat loss and fragmentation, the type and the strength of natural selection might change, favoring different kinds of life histories in fragmented than in continuous landscapes [8]. Populations may adapt to fragmented habitats and thereby compensate, to some extent at least, for the adverse demographic consequences of habitat loss and fragmentation [9],[10]. For instance, species living in newly-fragmented landscapes may evolve a lower [11] or a higher rate of dispersal [12], depending on the opportunity to establish new populations, temporal variation in population sizes, the cost of dispersal, and other factors [13]. However, the genetic basis of such adaptations is poorly understood and so far no genome-wide studies have been reported in this context.

**Figure 1:**
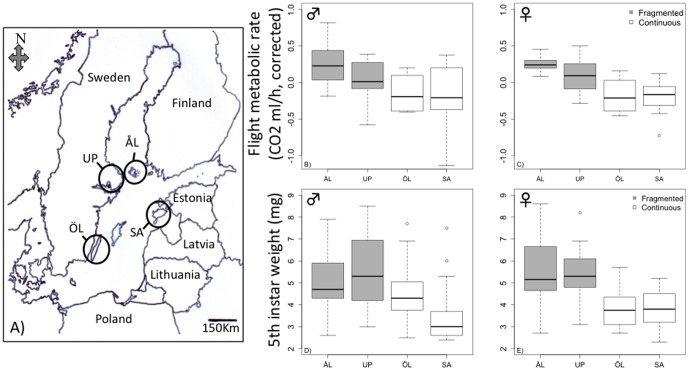
The four study populations in northern Europe and results on key life-history traits. (a) Map of the northern Baltic region with the locations of the four regional study populations: the Åland Islands (ÅL) in Finland, the Uppland coastal region (UP) in Sweden, the Saaremaa island (SA) in Estonia, and the Öland island (ÖL) in Sweden. (b) to (e), Peak flight metabolic rate (CO_2_ production, ml/h, corrected for variation in body weight) during active flight in males (b) and females (c), and the weight of the 5^th^ larval instar following winter diapause (mg) in males (d) and females (e). In panels (b) to (e), populations from fragmented landscapes are shown with gray shading, populations from continuous landscapes with open box. In ANOVA of pooled data for the two sexes, the effects of sex, population nested within the landscape, and family nested within population were not significant for either flight metabolic rate nor larval weight, but the effect of the landscape type was highly significant: *P* = 3.9e-05 for flight metabolic rate and *P* = 8.6e-12 for larval weight. For more detailed analyses of these and other life-history traits see [19].

The Glanville fritillary butterfly (*Melitaea cinxia*) metapopulation in the Åland Islands in Finland, inhabiting a network 4,000 small meadows, is a model system for the study of the ecological, genetic and evolutionary consequences of habitat fragmentation [6],[12]. For instance, previous studies have produced conclusive evidence for the presence of an extinction threshold in the demographic dynamics [14], while inbreeding depression in small local populations increases their risk of extinction [15]. Strong reciprocal effects have been detected between the extinction-colonization dynamics and allele frequency dynamics in a non-synonymous SNP (Single Nucleotide Polymorphism) in the coding region of the gene phosphoglucose isomerase (*Pgi*) [12],[16]. These studies highlight, from different perspectives, the key roles that local extinction and re-colonization play in metapopulations in highly fragmented landscapes. At the level of individuals' life histories, female butterflies from newly-established local populations are more dispersive than butterflies from old local populations [17], suggesting that local extinctions and re-colonizations select for an increased rate of dispersal in highly fragmented landscapes. Indeed, butterflies from more fragmented landscapes have higher flight metabolic rate (Fig. 1b-c) and, in support of model predictions [10],[18], are more dispersive in the field than butterflies from less fragmented landscapes [12],[13]. There are also other life history differences between populations inhabiting fragmented *versus* continuous habitats [19], for instance in the rate of larval development (Fig. 1d-e).

Here, we use individually tagged whole-transcriptome shotgun sequencing (RNA-seq) data from Glanville fritillary populations originating from four separate landscapes, two of which are highly fragmented and two of which are continuous landscapes. We characterize genetic differentiation among these populations in terms of allelic (SNP) and gene expression variation under common garden conditions. Our aim is to identify genetic variation that characterizes butterflies inhabiting fragmented *versus* continuous landscapes, which variation may underpin the life history differences that have been described for the respective populations. To supplement the results on the contrast between the landscape types, we bring into the analysis two types of additional information. First, in the well-studied Åland metapopulation in Finland [6],[12], which represents a fragmented landscape, we distinguish between butterflies originating from newly-established (the generation following colonization) *versus* old local populations, and correlate expression differences between these population types with the contrast between the landscape types. This is relevant, because high rate of population turnover, extinctions and re-colonizations, characterizes metapopulations in fragmented but not in continuous landscapes. Second, given the great significance of flight capacity for the dynamics of butterflies in fragmented landscapes, we compare the change in gene expression following an experimental flight treatment with the contrast in base-line gene expression between the fragmented and continuous landscapes. Our results, including expression patterns in the different data sets and the functional annotation of the respective genes, support the hypothesis of genomic adaptations to landscape fragmentation.

## Materials and Methods

### Sampling and rearing of butterflies

The material was collected in autumn 2009 as diapausing larvae from four regional populations (Fig. 1a). The Åland Islands in Finland and the coastal area in Uppland, Sweden, represent fragmented landscapes, where individual habitat fragments (dry meadows) are typically <<1 ha in area (median 0.17 ha for 4,000 meadows in Åland [20]). In contrast, on the large islands of Öland in Sweden and Saaremaa in Estonia (Fig. 1a), the suitable habitat occurs mostly in large continuous areas, called calcareous ‘alvar’ grasslands, often exceeding 100 ha in area [21]. Glanville fritillary is not protected in any of these regions and no permits were needed for sampling of larvae. In each regional population, four larvae were collected from 50 different families located across >100 km^2^, though only one larva per family was used for sequencing. The larvae overwintered at +5°C and 85% RH. In spring 2010, larvae were reared singly under common garden conditions (12∶12 L/D; 28/15°C) with fresh leaves of the host plant *Plantago lanceolata*. Adults were held in the same conditions for three days. Samples were taken at 11 am by cutting half of the abdomen for a DNA sample and placing the rest immediately into liquid nitrogen for an RNA sample (Table S1). Samples were stored in −80°C.

### Reference genome and genomic methods

The genome of the Glanville fritillary consists of 8,262 scaffolds, of which 72% have been located in 1,453 superscaffolds (Ahola et al. in prep.). Altogether 16,667 gene models (v1.0) were predicted using a combination of *ab initio* and evidence-based methods in MAKER [22]. While building the gene models, we used protein sequence information from other species and full-length transcriptome sequencing of the Glanville fritillary as evidence. Functional annotation was obtained for 12,410 gene models using the PANNZER annotation tool [23], which gave a GO category for 9,471 genes. A KEGG category was obtained for 3,685 genes using the KAAS annotation server. Based on a linkage map [24], 94% of all predicted genes are located in scaffolds with existing chromosome information.

### RNA-seq library preparation, sequencing, filtering and mapping

Total RNA was extracted from frozen butterfly thorax with Trizol (Life Technologies Corporation, CA, USA) followed by chloroform, phenol-chloroform-isoamylalcohol and second chloroform purification. RNA was precipitated with isopropanol and washed with ethanol. RNA quality was verified with NanoDrop measurement (Thermo Fisher Scientific Inc., MA, USA) and Bioanalyzer run (Agilent Technologies, CA, USA). An in-house library construction protocol was designed to enable large-scale sample processing with relatively low cost. PolyA-anchoring based libraries were designed to simplify the wet-lab protocol and to reduce complexity of read-count data, and wide fragment size distribution was utilized to increase the number of coding exon variants covered. 5µg of the total RNA was used in cDNA synthesis (RevertAid transcriptase, Thermo Scientific) with biotin linked PolyT primers (5′-Biotin-TTT TTT TTT TTT TTT TTT Tspacer-C-3T TdUT TVN-3′). Biotin linked cDNA was RNaseH treated and filter purified (Amicon Ultra 30K, Millipore). Purified cDNA product was attached to streptavidin beads (DynaBeads M-270 Streptavidin, Invitrogen, Life Technologies) and washed and pre-annealed 5′adapters were ligated (5′-ACA CTC TTT CCC TAC ACG ACG CTC TTC CGA TCT NNN NNN-3′, 5′-Phosphate-AGA TCG GAA GAG CGT CGT GT-3′). After second-strand synthesis with Klenow fragment (Thermo Fisher Scientific Inc.) DNA product was released from streptavidin complex (USER enzyme, New England Biolabs) followed by pre-annealed PolyA adapter ligation (5′-GTG ACT GGA GTT CAG ACG TGT GCT CTT CCG ATC TTT-3′, 5′-Phosphate-GAT CGG AAG AGC ACA CGT CT-3′). Released end product was then bead purified (Agencourt Ampure XP, Beckman Coulter Inc., CA, USA) before PCR amplification with indexed primers (5′CAA GCA GAA GAC GGC ATA CGA GAT XXX XXX GTG ACT GGA GTT CAG ACG TGT GC-3′, 5′-AAT GAT ACG GCG ACC ACC GAG ATC TAC ACT CTT TCC CTA CAC GAC GCT-3′). After 20 to 25 PCR cycles the amplified library was run on agarose gel and separated by size into two fractions, 300–500 bp and 500–800 bp, to control a possible transcript length associated bias. Semi-automated size separation was performed with carboxy beads (Nordiag AB, Sweden) and Magnatrix1200 Biomagnetic Workstation (Nordiag) as previously described [25]. After size separation concentrations were individually measured with Qubit fluorometer (Life technologies) and 12–24 samples were pooled with equimolar amounts to separate 300–500 bp and 500–800 bp pools, and the library quality was verified with a Bioanalyzer run and Qubit measurements.

RNA-seq libraries were sequenced in Karolinska High Throughput Center (Sweden) and in the DNA sequencing and Genomics laboratory, the Institute of Biotechnology (Finland) according to the manufacturer's instructions in insert size-specific lanes with Illumina HiSeq2000 and HiScanSQ (Illumina Inc.,CA, USA), with only the 5′ end of each fragment being sequenced with 75 or 100 bp read length. Read trimming was done by finding the longest subsequence with Phred-score >20 for all bases, after which poly-A was trimmed (in-house C program). The threshold for minimum read length was 50 bp. Read mapping was done using TopHat2 [26] against genomic scaffolds. Statistics on sequence reads are given in Table S2 and in Fig. S1. Due to differences in sample size per population (Table S1) and the number of sequence reads per individual (Fig. S1), we created a balanced dataset by including 15 individuals per population with the largest number of sequence reads (minimally 3M mapped reads per individual). The entire material consisted of 174 individuals from the four populations, including some individuals with <2M reads per individual.

### SNP detection

SNPs were detected from RNA-seq read alignments using in-house software. Because we used unnormalized RNA-seq libraries, there are several low-coverage regions in the transcriptome. SNPs were called from uniquely mapped reads in which the base call quality was above 20 (Phred score). Candidate SNPs were first selected from the balanced set of 4×15 individuals (15 individuals from 4 populations), requiring that the SNP was biallelic in the pooled data of 174 individuals. The set of 243,019 SNPs thus obtained was further restricted to 103,606 SNPs, requiring that there were at least ten individuals per population and that the global minor allele count was at least nine (5% of the 174 individuals). We experimented with different minimum coverage thresholds between 1 and 10 and ascertained that allele frequency differences between the populations were robust, in other words remained the same for the different thresholds. Differences between the populations were similar using both individual-based and population-based approaches. Similarity of individuals was investigated by hierarchical clustering and Sammon mapping [27].

### Allele frequency analyses

**Figure 2:**
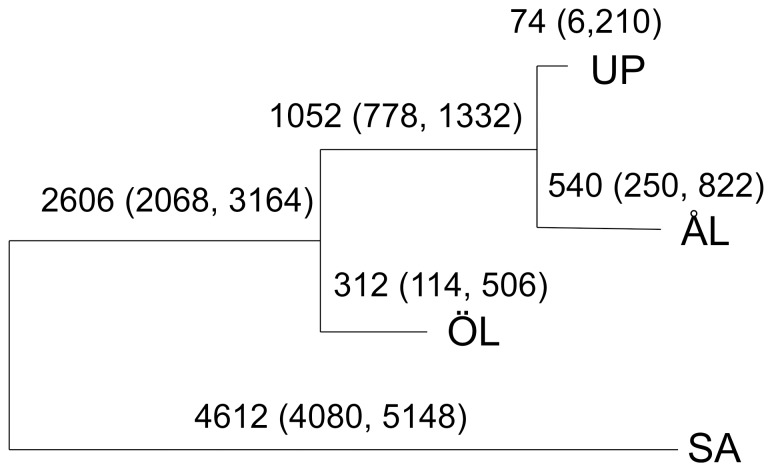
Genealogy of the four study populations. Branch lengths (also given as numbers: expectation and 95% posterior probability interval) represent divergence times in years. The branch lengths are measured on the scale *t/2N_e_*, where *t* is the number of generations (here one per year) and *N_e_* is the effective population size. *N_e_* = 10^4^ was assumed for all populations (Materials and Methods).

The statistical significance of allele count differences was calculated using Fisher's exact test. Due to multiple loci, *P*-values were converted into False Discovery Rate (FDR) values. Each population pair was compared against each other to find out which results are specific to a pair of populations and which results are due to a single population. Differences in the numbers of population-pair specific SNPs are highly significant (Fig. 2; 4.8e–275 after Bonferroni correction). In addition to Fisher's exact test, we performed permutation tests to find significant differences between the populations from fragmented *versus* continuous landscapes. This test resulted in 12,227 SNPs with FDR<0.05 out of the 103,606 candidates. Applying Bonferroni correction instead of FDR, there were 5,396 SNPs with adjusted *P*-value<0.05. In contrast to these numbers, Fisher's exact test is more conservative, and it resulted in 4,389 SNPs with FDR<0.05. 4,012 (91%) of the latter SNPs had FDR<0.05 in the permutation test.

### Within-population genetic diversity

We examined the numbers of polymorphic SNPs in the four regional populations. Since sample size varied between the populations, we sampled 10 individuals from each population (without replacement) 100 times. In each sample of 4×10 individuals, populations were ranked based on their diversity. UP was ranked first 92 times, ÖL the second 80 times, ÅL the third 55 times, and SA the fourth 63 times. We repeated the calculation for different allele detection thresholds from one to three read counts, which resulted in the same ranking of the populations. Secondly, we calculated the number of different SNP genotypes divided by the number of shared SNPs between each pair of individuals. The order of the populations was the same as with the first method, variability being highest in UP. A SNP was scored heterozygous if at least three reads were detected for both alleles, and homozygous if only one allele was detected with read count greater or equal to 10. In other cases the SNP was excluded.

The genome-wide allelic variation shows that the four regional populations are closely related to each other, and the COI sequence (A. Duplouy, unpublished data) shows that they belong to the eastern (Asian) clade as defined by [28].

### Population genealogy

The well-studied metapopulation in ÅL has had a census population size between 2×10^3^ and 2×10^4^ in terms of larval family groups in the past 20 years [12], which roughly corresponds to a *N_e_* of 10^3^ to 10^4^ in terms of adult butterflies (larvae live gregariously in sibling groups of 100 larvae). Given that the amount of habitat has somewhat declined over the past 100 years, a reasonable single figure for *N_e_* is 10^4^. There are no census data for the other study areas in Fig. 1a, but they all have comparable or somewhat greater total areas of suitable habitat than ÅL, and we assume that *N_e_* is of the order of 10^4^ also for these regional populations.

The Bayesian drift model BANANAS [29] was fitted to several random subsets of SNPs (of the size 500, 500 and 1000 SNPs, respectively) to ensure that congruent estimates of population genealogies were obtained. For each subset, estimation was repeated four times to assess convergence of the adaptive Monte Carlo algorithm, using the parameters *N_0_* = 10000, *T* = 10, *N_t_* = 1000; *N_0_* = 5000, *T* = 10, *N_t_* = 500; *N_0_* = 6000, *T* = 10, *N_t_* = 600 [29]. Since the number of observed populations is small, all possible tree topologies were considered enumeratively and the same maximum *a posteriori* genealogy had approximately the probability 1.000 for each SNP set. There was some variation in the estimated branch lengths across the different SNP sets, but this did not change the interpretation of the results (data not shown). The software package BAPS v5.4 [30],[31] was used to estimate the population genetic structure underlying the data. Based on data for 103,606 SNPs (above) we used 10 estimation runs for the individual clustering model with the prior upper bound for the number of populations in the range [2],[15]. The posterior approximation was highly concentrated on *k* = 2 clusters. The cluster that contained more than one population was re-analyzed separately using a similar approach, which again resulted in *k* = 2 clusters, based on the 100,808 SNPs that remained variable. Such a hierarchical Bayesian clustering approach has been shown to considerably enhance the detection of more subtle boundaries in gene flow in data where some subpopulations are markedly distinct from the others [32],[33].

### Bayesian clustering of populations

Similarity of the populations in terms of their gene expression profiles was assessed by computing the marginal likelihoods of all possible population combinations, i.e. partitions obtained by combining one or more of the populations together. There are 15 different such partitions (including the one where no populations are combined, but each one forms its own cluster). The data was preprocessed with TMM (trimmed mean of M values), then log-transformed and finally each gene was normalized to zero mean and unit variance over the whole population. For each cluster and each gene, the marginal likelihood was computed for a Gaussian model N(*m*, 1/*p*), whose precision parameter (*p*) is distributed as Exp(1), and whose mean parameter (*m*) is distributed as N(0, 1/*p*). Among the 15 partitions, the one where all four populations are separate had the greatest marginal log-likelihood (−3.9715 * 10^−6^). Almost as large a log-likelihood (−3.9748 * 10^−6^) was obtained by combining ÖL and UP together. Other partitions were clearly inferior, and the lowest log-likelihood (−4.1077 * 10^−6^) was obtained when all populations were combined into one.

### Gene expression analyses and statistical tests

Populations were compared based on their gene expression profiles. Read counts were normalized according to the TMM method [34]. The average level of expression was close to zero for half of the 16,667 gene models in all populations, while the expression of a few genes was very high (Fig. S2). Differentially expressed genes were identified using edgeR package [35],[36] and a permutation test. EdgeR assumes negative binomial distribution for normalized read counts. Each gene had its own dispersion parameter. A generalized linear model was fitted with four parameters, one for each population mean. A contrast function was defined as the difference between the two fragmented and two continuous populations, and its statistical significance was tested with the likelihood ratio test. A permutation test (implemented in R) was used to identify differentially expressed genes between the fragmented and continuous populations using logarithmic normalized read counts. We required that population means for both fragmented populations were higher/lower than population means for the two continuous populations to calculate the significance of the difference. The *P-*value was calculated based on 10^5^ permutations for each gene and converted into a FDR value. Finally, we required that differential expression was statistically significant in both edgeR analysis and by the permutation test, which resulted in 1,841 genes.

Gene Ontology enrichment analysis was performed using the topGO package in R. Since our RNA-seq reads were anchored at the 3′ end of gene (see RNA-seq library preparation), differences in gene lengths do not bias the results unlike in traditional whole-length transcript libraries [37]. In the KEGG enrichment analysis we used Fisher's exact test implemented in R. Publication enrichment was conducted with the FlyMine service (v.36.1 [38]) using *Drosophila melanogaster* orthologs. *Wnt*, *Notch* and *Hedgehog* signalling pathways for *D. melanogaster* were retrieved from KEGG and used to find ortholog *Melitaea cinxia* genes using blastx (with a minimum bitscore value of 100).

eQTL analyses relating genotype to gene expression were conducted with gls function of the nlme package in R. The structure of the covariance matrix describing the relatedness of individuals was calculated based on shared alleles in 103,606 SNPs (see above). The *P*-values of the regression coefficients were converted into FDR values; we accepted all SNP-gene expression pairs in which FDR<0.05 in at least one population, while the sign of the regression coefficient was required to be the same in the remaining populations regardless of their significance.

### Heterogeneity in gene expression within populations

We examined within-population heterogeneity in gene expression running Bayesian mixture model (GMM) analysis using the VB-MOG algorithm [39]. This was done to justify subsequent analyses of the expression data. For each gene, GMMs with one to five mixture components were fitted and the best model was selected according to the highest marginal likelihood. Logarithmic TMM-normalized read counts were scaled to have zero mean and unit variance within each gene so that the same (uninformative) priors could be used for each gene. Mixture components were initialized with the *k*-means algorithm (implemented in R) and the VB-MOG algorithm (in-house C implementation) was applied until convergence. While investigating bimodal expression profiles, samples were classified into two categories based on the posterior probabilities of the mixture components. Note that although read counts were normalized between individuals, normalization does not completely remove sampling effect especially for low-expression genes.

In the sample of 49 individuals from ÅL, there were 8,360 genes with an average normalized log count greater than one. Out of these genes, the best model had 1, 2 and 3 mixture components in 6,929, 1,383 and 48 genes, respectively (none had >3 components). Similar results were obtained with pooled data from all four populations (selecting 4×15 = 60 individuals that had least missing data). Since the vast majority of expression distributions are best explained by a single Gaussian, we used standard statistical tests assuming unimodal distributions while comparing populations below. We examined more closely the set of 1,383 genes for which the best model had two mixture components in ÅL. First, we tested for a possible association between mixture components and gender. There were 663 genes with a significant gene expression difference between males and females (FDR<0.05), but out of these genes, only 68 showed a significant (FDR<0.05) association between the two mixture components. Second, the distinction between new and old local populations (below) did not correspond with the two mixture components in any of the genes. Third, there were only a few significant associations between the mixture components and alleles (results not shown). Given these largely negative results, we conclude that two mixture components can often be the result of insufficient RNA-seq coverage in the gene.

### Flight experiment

The full description of the experiment and the results are available in GEO (accession number GSE47942) and reported in Kvist et al. (in prep.). The butterflies originated from the Åland metapopulation. Butterflies were encouraged to fly actively in a metabolic chamber for 15 mins, which was long enough to make many of them completely exhausted. RNA was sampled at 1 and 20 hours after the flight treatment (*n* = 15 in each case) and from control individuals (no flight treatment, *n* = 12) and sequenced in a similar manner than in the present study. The analysis is focused on the 39 genes with a significant expression difference in both males and females between individuals sampled at 20 hours after the flight treatment *versus* control individuals.

### Data deposition

The data have been deposited in NCBI's Gene Expression Omnibus [40] and are accessible through GEO Series accession number GSE47692 (http://www.ncbi.nlm.nih.gov/geo/query/acc.cgi?acc=GSE47692).

## Results

### Post-glacial colonization history and genetic population structure

We used the Bayesian drift model BANANAS to estimate population genealogies with genome-wide SNP data, from which a random subset was chosen (500 SNPs; see Materials and Methods; sample sizes in Table S1). The branch lengths in Fig. 2 are measured on the scale *t/N_e_*, where *t* is the number of generations (one per year) and *N_e_* is the effective population size. Assuming *N_e_* = 10^4^ (Materials and Methods) gives the divergence time estimates shown in Fig. 2. Saaremaa (SA) has diverged from the other populations of the order of thousands of years but <10,000 yrs ago; the Öland (ÖL) population diverged from the Åland Islands (ÅL) and Uppland (UP) populations several hundred years ago; while the ÅL and UP populations have diverged more recently. These estimates are consistent with the known post-glacial colonization from the east [28] and the presence of water barriers that had to be crossed to reach ÅL, UP and ÖL (Fig. 1a), possibly with unintentional human assistance. The close affinity of ÅL and UP may reflect infrequent gene flow since the colonization of UP from ÅL. We presume that ÖL has been colonized along the Swedish coastline from the north (Fig. 1a). Analysis of the spatial genetic structure of the four populations with BAPS v5.4 gave congruent results (Fig. S3). Figure S4 shows that UP has the highest within-population genetic diversity, followed by ÖL and lastly by ÅL and SA. The greater genetic diversity of UP may be due to its mainland location and previously (though not currently) more extensive distribution and possibly greater abundance of the butterfly (Fig. 1a).

### Allelic and gene expression variation among landscape types

**Figure 3:**
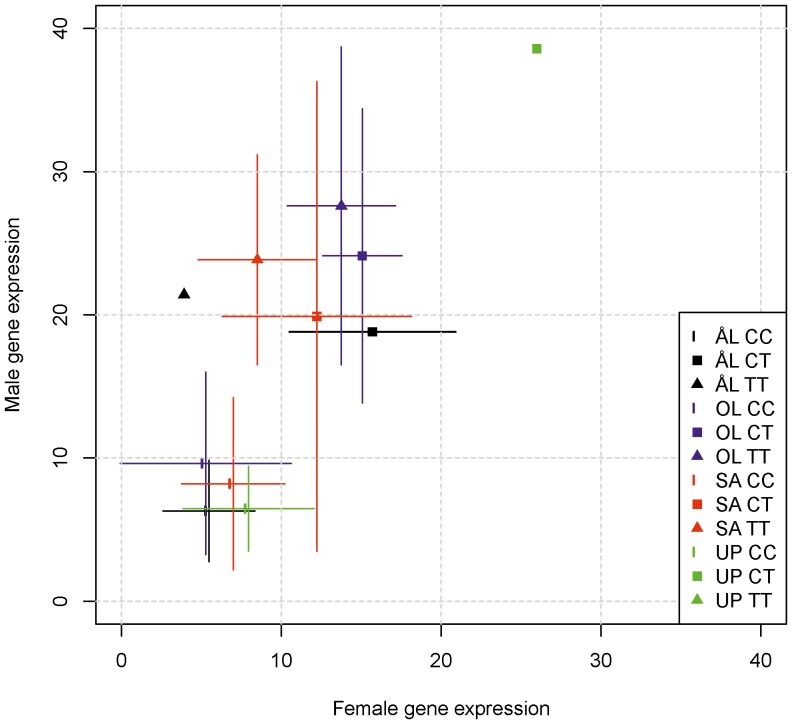
Clustering of the four study populations based on allele frequencies (a) and logarithmic gene-specific read counts (b). Distance was defined as one minus correlation. The lower panels show the Venn diagrams for the number of SNPs between pairs of populations with statistically significant (FDR<0.05) allele frequencies (c; Fisher's exact test) and for the number of statistically significant (FDR<0.05) differentially expressed genes (d; edgeR analysis). The pairs of populations representing the contrast between fragmented *versus* continuous landscapes are shown by gray shading.

We examined the relationships of the four populations in terms of allelic and gene expression variation, with the particular aim of contrasting the two fragmented populations (ÅL and UP) with the two continuous populations (SA and ÖL). In clustering of the four populations based on their allele frequencies, ÅL and UP are the two most similar populations and SA is the most distinct one (Fig. 3a), essentially replicating the result in Fig. 2 based on a different model. We compared allele frequencies among the populations for each SNP, contrasting all pairs of populations with each other, one contrast corresponding to fragmented *versus* continuous landscapes. The numbers of SNPs with statistically significant differences between population pairs are shown in Fig. 3c. The pairing corresponding to fragmented *versus* continuous landscapes shows many more SNPs (5,925) with a significant difference than the other pairings (163 and 1,996). However, an obvious confounding factor here is the colonization history of the populations, which may largely explain the contrast in allele frequencies between the ÅL+UP *versus* SA+ÖL pairings.

Gene expression differences among the four populations were analysed using a balanced data set with 15 individuals from each population (Materials and Methods; Table S2). The relationships between the four populations based on gene expression are different from those based on allelic variation. SA is the most distinct population in both analyses, but in gene expression data ÖL and UP cluster together with a clear contrast to ÅL (Fig. 3b). This result was supported by Bayesian clustering of populations (Fig. S3). In the comparison of pairs of populations, the contrast between the fragmented (ÅL and UP) *versus* continuous populations (SA and ÖL) showed the largest number of significant expression differences (Fig. 3d; 2,643 SNPs in 2,567 different genes). This result, unlike the result for allelic variation, cannot be parsimoniously explained by common ancestry, because in the expression data UP and ÖL cluster most closely (Fig. 3b).

We used an enrichment analysis based on the GO and KEGG gene classes to investigate differences in expression level between the landscape types (Table S3). Based on a permutation test and a likelihood ratio test, we identified a set of 1,841 genes with differential expression between the landscape types (Table S4). Altogether 178 GO categories were significantly enriched (107 categories for biological process, 31 for cellular component, 40 for molecular function), including multiple categories involved in glucose import, growth and differentiation, hormonal signalling, immune response, transcription, RNA processing and translation. Processes that potentially reflect adaptation to different landscape types include many stimulus response GO groups (chemicals, hormones and temperature), cellular response to misfolded and unfolded protein, RNA splicing and growth. Many categories related to growth and differentiation contain genes involved in *Wnt, Notch* and *hedgehog* signalling pathways, which is also indicated by publication enrichment against *Drosophila* [41],[42]. A significant proportion of these signalling pathway genes were differentially expressed between the fragmented *versus* continuous landscapes (14/93 for *Wnt*, 9/35 for *Notch* and 19/95 for *hedgehog* signalling pathways; Table S5). Overall, extracellular signalling was up-regulated in samples from fragmented landscapes, including the main signalling molecules *Wnt* and *hedgehog*, whereas intracellular signalling in the *hedgehog* pathway was consistently down-regulated, starting from *hedgehog* receptor *patched*. The KEGG enrichment found only four significantly enriched categories: riboflavin metabolism, lysine degradation, primary bile acid biosynthesis and other types of O-glycan biosynthesis.

### Elevated hexamerin expression in fragmented landscapes

Previous studies of the Glanville fritillary have linked faster larval post-diapause development [43] and increased female reproductive capacity [44] to elevated expression of hexamerin (larval serum protein) genes. Since the rate of larval post-diapause development is faster in fragmented than continuous landscapes (Fig. 1d,e), we specifically looked at the expression of hexamerin genes in our analysis. Of the seven known hexamerin genes annotated for the Glanville fritillary, four genes (MCINX010010, MCINX015566, MCINX015567, MCINX015572) had a significantly (edgeR FDR<0.05) higher expression in fragmented landscapes. The probability of having 4 or more significant hexamerin genes among the 7 genes is 0.0039 (from the binomial distribution, given that the frequency of genes with a significant difference between the landscape types among all the genes is 0.11 (1841/16667)). Additionally, multiple hexamerin modifying genes in the ecdysteroid signaling pathway were differentially expressed between the landscape types. Among the known downstream genes of hexamerin expression [45], a substantial fraction of cuticle protein (14/93) and troponin (2/6) genes had higher expression in populations from fragmented landscapes. All but one of the cuticle protein genes (14/15) that showed a significant expression difference between the landscape types had higher expression in fragmented landscapes.

**Figure 4:**
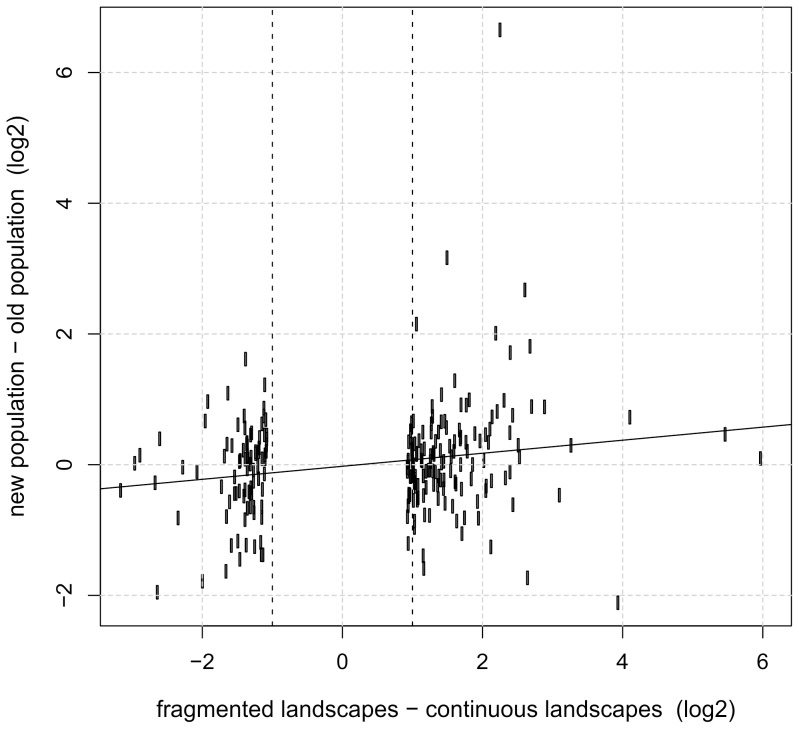
Gene expression in new *versus* old local populations and fragmented *versus* continuous landscapes. Differential expression of 233 genes (Table S6) in females from newly-established *versus* old local populations in the fragmented landscape in Åland (vertical axis) against differential expression of the same genes in fragmented *versus* continuous landscapes in three populations (horizontal axis; only UP represents fragmented landscapes to have independent data sets on the two axes). Correlation is significant (*P* = 0.0031).

Fragmented landscapes harbor metapopulations that consist of small local populations connected by dispersal [6],[12]. As local extinctions and re-colonizations are predicted [10] and observed [13] to select for more dispersive females, we hypothesize that expression differences between butterflies from new *versus* old local populations within a single fragmented landscape are correlated with expression differences between fragmented *versus* continuous landscapes. To test this hypothesis, we examined expression differences in female butterflies between fragmented *versus* continuous landscapes with the expression difference between females originating from newly-established (*n* = 6) *versus* old local populations (*n* = 18) in the fragmented ÅL landscape. For this comparison, we selected the 1,841 genes with a significant expression difference between the landscape types. We filtered these data to retain the genes with adequate coverage in both data sets (logCPM>1), and we further retained only genes with a large expression difference between the landscape types (|log_2_ count ratio|>1), leaving 254 genes. In these data, there is a highly significant relationship in the expected direction between the expression difference between the landscape types and the expression difference between the new *versus* old local populations (*P* = 0.00018). To make certain that this relationship was not due to butterflies from ÅL being included in both variables, we re-calculated the expression difference between the fragmented and continuous landscapes after excluding ÅL butterflies, leaving 233 genes (Table S6). The result remained qualitatively the same (Fig. 4; *P* = 0.0031).

### Association between SNP alleles and gene expression

**Figure 5:**
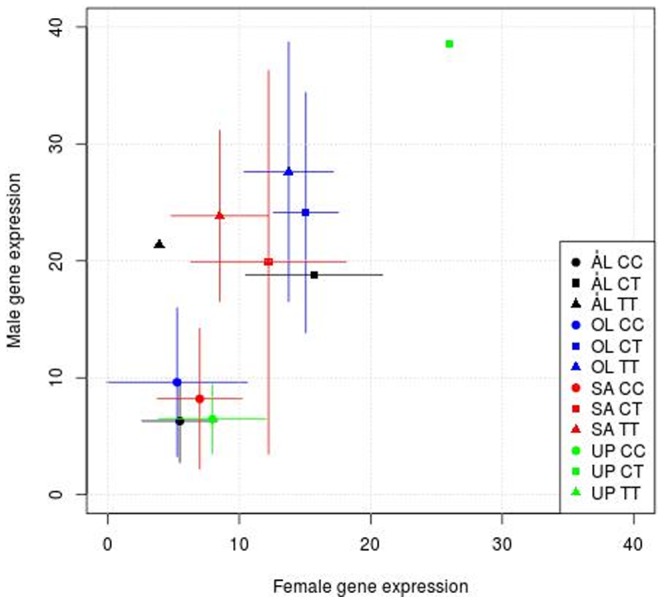
Association of gene expression with SNP polymorphism in *Hif-1a.* The figure shows expression of the hypoxia-inducible factor 1 alpha (*Hif-1a*) in males (vertical axis) and females (horizontal axis) in the four populations, separately for the three genotypes CC, CT and TT in the SNP MCINX007807. The figure gives the means and standard deviations for normalized read counts. In a linear model, the effect of genotype is highly significant (TT compared with CC, *P* = 1.7e-12, CT compared with CC, *P* = 4.6e–12), and the sex * genotype interaction is significant (TT, *P* = 0.009, CT, *P* = 3.9e–05). Adjusted *R^2^* = 0.49.

To search for genomic regions which are likely to harbor intragenic or proximate allelic variants affecting transcript levels, we investigated associations between SNPs and gene expression (*cis*-eQTL) with regression models assuming additive, dominance and recessive effects. Each SNP was tested to explain expression of its closest gene. To reduce spurious associations, we required that all three genotypes (the two homozygotes and the heterozygote) were represented in the data and that the analyses could be done separately for each population. Only those SNPs that had the same sign of the association in all four populations were included. We focused on SNPs that are potentially biologically significant for landscape adaptation, and we hence required that there was a significant allele frequency difference between the fragmented and continuous landscapes. With these criteria, we arrived at 70 SNP-gene expression pairs in 60 genes (Table S7). SNPs located within predicted exons were recorded as synonymous or non-synonymous, but most SNPs were outside the predicted gene models (in 3′ UTR or in missing exons due to incomplete gene models). The 60 genes included genes that are important for hypoxia response (hypoxia-inducible factor 1 alpha, *Hif-1a*, MCINX007807) and genes involved in *Notch* (*Nicastrin,* MCINX013215) and *hedgehog* (*Patched-related,* MCINX011439) signaling pathways (Table S7). SNPs in these genes are promising candidates for large-scale genotyping to study interactions between genotype, gene expression, life history traits and their interactions with environmental variables. As an example, we highlight in Fig. 5 *Hif-1a,* which has a key function in the response to hypoxia. There is a striking genotypic effect on expression, which is consistent across all four populations, and there is a significant allelic difference between the fragmented (frequency of the CC genotype in ÅL: 0.83, UP: 0.91) and continuous landscapes (SA: 61, ÖL: 0.51; *P* = 0.0003 for the difference between the landscape types). These results reflect possible landscape-level selection for increased hypoxia tolerance in the more mobile butterflies in fragmented landscapes. Finally, there is a significant sex-genotype interaction, males showing higher expression for the high-expression genotype (Fig. 5).

### Flight-induced gene expression and expression difference between the landscape types

**Figure 6:**
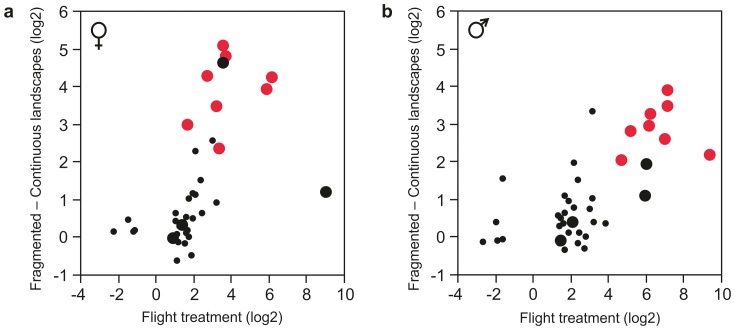
Change in gene expression following active flight compared with the expression difference between the landscape types. The horizontal axis gives the change in expression following experimental flight treatment, while the vertical axis shows the expression difference between the fragmented *versus* continuous landscapes (without the flight treatment) in females (a) and males (b). The data set includes the 39 genes (Table S8) in which expression at 20 hours after the flight treatment was significantly and similarly different from the control individuals in both females and males. The large symbols indicate 12 genes with immune response functions, of which 8 genes are AMPs (in red) (Table S8). The 12 immune response genes (but not the remaining 28 genes) are more strongly up-regulated in males than in females following flight (*P* = 0.01).

Here, we used the results of an experiment on gene expression changes following active flight to test whether the flight-induced expression changes are correlated with the base-line expression difference (no flight treatment) between the landscape types (Materials and Methods). We focus on the 39 genes that were significantly up-regulated (34 genes) or down-regulated (5 genes) in both males and females at 20 hrs following the flight treatment (Materials and Methods, Table S8). Figure 6 shows that the genes that are more highly expressed in fragmented than continuous landscapes are significantly up-regulated following the flight treatment. The correlation is highly significant, but we refrain from presenting a *P*-value, because the degrees of freedom are inflated to an unknown extent by the fact that the expression of many genes is regulated by the same factors. The most noteworthy result is the abundance of immune response genes among the 39 genes, specifically antimicrobial peptide (AMP) genes such as *gloverin*, *moricin*, *defensin*, *lebocin* and *cecropin* genes (Table S8).

## Discussion

A large number of genes were differentially expressed between the populations when tested in common garden conditions, and 11% of all the genes (1,841/16,667) were differentially expressed between populations inhabiting fragmented *versus* continuous landscapes. We suggest that these differences reflect dissimilar selection in dissimilar environments and are causally related to the known life history differences between the two landscape types. Admittedly, we cannot conclusively reject alternative explanations because of several confounding factors, which is an inevitable challenge in observational studies conducted at large spatial scales. First, the two fragmented landscapes are located at a slightly higher latitude than the two continuous landscapes (Fig. 1a), though the difference is small and there is only marginal difference in their climates [19]. Second, the two fragmented populations share the most recent common ancestor, as evidenced by genome-wide allele frequencies (Fig. 3a,c), but note that the pattern of differentiation is different in gene expression (Fig. 3b,d), suggesting that gene expression does not merely reflect common ancestry. Third, the continuous landscapes (SA and ÖL) have flat limestone bedrock, which is the reason for the continuous grassland habitat, but all the four study regions have the same larval host plants and there are no known differences in habitat quality that would affect the butterfly populations. On the other hand, several features in our results support the hypothesis that landscape fragmentation is one cause of gene expression differences. First, there were significant expression differences between the landscape types in several categories of genes that are likely to be related to the known life history differences between the landscape types, for instance the hexamerin genes and other categories related to growth and regeneration (discussed below). Second, genes that had higher expression in newly-established than in old local populations within the fragmented Åland landscape had higher expression, at the landscape level, in the more fragmented landscapes (Fig. 4). This correlation suggests that recurrent establishment of new populations by dispersing females in fragmented landscapes [12] selects for a specific gene expression profile. And third, genes that were systematically up-regulated following an experimental flight treatment tended to have higher base-line expression in fragmented than continuous landscapes (Fig. 6), suggesting that butterflies in fragmented landscapes are genetically primed for a high level of flight activity. Note that the latter two results, which are further discussed below, involve relationships between independent data sets.

### Larval growth rate and adult reproduction

Genes involved in growth and regeneration and which have significant expression differences between the populations in the present study include many members of the pleiotropic *Wnt*, *Notch* and *hedgehog* signalling pathways. Two genes (*Nicastrin* and *Patched-related*) in these pathways showed significant associations between allelic variation and gene expression, suggesting possible selection. These pathways have been shown to be activated during regeneration of induced wing disk tissue in *Drosophila* [41],[46], which could be related to the ability to recover from flight-induced oxidative damage to flight muscle tissue [47]–[51]. Genes such as *Socs2* (MCINX002283), *Chitinase-like 3* (MCINX013513) and *Tenectin* (MCINX005469) had higher expression in populations from fragmented than continuous landscapes, and they are up-regulated following a flight treatment (Kvist et al. in prep.).

A notable life history difference between the fragmented and continuous landscapes is faster larval development in the former [19], related to greater post-diapause body size (Fig. 1d,e). This could be caused by differences in energy reserves received by the offspring from their mothers, but equally there may be genetic or epigenetic differences in hormonal regulation, influencing the development time. In particular, we found systematic differences in the expression of hexamerin genes between the landscape types, which have previously been shown to have high heritability and which correlate strongly with larval development time in the Glanville fritillary [43]. The expression of hexamerin genes is mediated by ecdysteroid hormones (particularly 20-hydroxy-ecdysone, 20E), and it varies among families within the metapopulation in Åland [44] and exhibited significant differences between the fragmented and continuous landscapes in the present study. The co-expression of hexamerin genes and the hexamerin modifying genes in the ecdysteroid signaling pathway suggests that the expression of these genes is driven by differences in hormonal regulation. Our samples represent adults rather than larvae, but differences in the regulators of hexamerin expression (promoter regions, enhancer elements and transcription factors) are likely to affect expression levels in all life history stages.

During their adult life, Glanville fritillary butterflies lose up to 30% of their thorax weight [52]. The resources thereby released are converted from fat body and muscle tissue to a soluble form of larval serum proteins (that is, hexamerins) [53],[54], which are carried through the hemolymph to the abdomen and used for reproductive organs [55],[56]. Importantly, the decline in thorax mass is greater in reproducing than in non-reproducing females [52], indicating that females use resources in the thorax for reproduction. This process is coordinated by hormonal regulation via ecdysteroids produced in the reproductive organs and, in larvae, the prothoracic glands [57]. Apart from differences in hexamerin genes, the angiotensin-converting enzyme (*Ace*), which regulates oviposition in Lepidoptera, has higher rate of expression in new than old local populations [44] and in fragmented than continuous landscapes (in the present study, FDR = 0.0001). These results are consistent with faster egg maturation in females in new than old local populations [44].

### Flight-induced expression changes and expression difference between the landscape types

Butterflies from fragmented landscapes have higher flight metabolic rate than butterflies originating from continuous landscapes (Fig. 1b,c), consistent with models [13],[18] predicting selection for high dispersal capacity in highly fragmented landscapes in the Glanville fritillary. Within fragmented landscapes, frequent re-colonizations select for high flight metabolic rate [58] and dispersal rate [17]. Flight is necessary for dispersal and re-colonization but flapping flight, such as butterfly flight, is an energetically expensive form of locomotion [59]. In flying insects, flight metabolic rate may exceed resting metabolic rate by two orders of magnitude, and flight muscle accounts for most of the whole-body oxygen consumption [60]–[62].

Most Glanville fritillary butterflies become exhausted within less than 15 mins of active flight (for examples of continuous records of CO_2_ output see [44],[63]). Butterflies may run out of fuel, but equally or even more likely they run out of oxygen, as hypoxia signaling has been reported to be activated during flight [64]. In the Glanville fritillary, allelic variation in the gene encoding for the enzyme succinate hydrogenase D (*sdhd*) has been associated with enzyme activity, flight metabolic rate and tracheal volume [64]. Significantly, butterflies with smaller tracheal volume exhibited higher hypoxia signaling following active flight [64]. Examining expression changes following active flight, Kvist et al. (in prep.) observed changes in the expression of the hypoxia-inducible factor 1-alpha (*Hif-1a*), a transcriptional activator that senses intracellular oxygen levels [65], as well as significant enrichment for hypoxia responsive genes and down-regulation of glycolysis and TCA. In the present study, the expression of *Hif-1a* was lower in populations in fragmented than continuous landscapes, which suggests that populations in fragmented landscapes are less sensitive to changes in oxygen level, allowing for higher peak metabolic performance during flight before the hypoxia response sets in. In *Drosophila*, an experimental evolution study selecting for hypoxia tolerance found an enrichment of genes in *Notch* signaling, and subsequent genetic testing using RNAi demonstrated that these genes indeed affect hypoxia tolerance [66]. In our study, genes in the *Notch* signaling pathway showed differences between the landscape types, and one gene (*Nicastrin*) exhibited significant association between allelic variation and expression (Table S7), possibly indicating selection.

Comparing the different landscape types, we found that the 39 genes that were significantly up-regulated following an experimental flight treatment tended to have higher expression in fragmented than continuous landscapes (Fig. 6). This result suggests that a set of genes that respond to active flight have higher basal expression in fragmented landscapes, in which butterflies fly more and have higher flight metabolic rate (Fig. 1b,c) than in landscapes with continuous habitat. One of the GO categories represented among the 39 genes is related to cellular response to misfolded and unfolded protein, including the heat shock cognate protein 70 (*hsc70*). This gene is activated in hypoxia [67] and in larval diapause [68], and it is involved in the recruitment of unfolded proteins for degradation [69]. *Hif-1a* has been reported to interact with *hsc70*, a member of a chaperone-mediated autophagy complex [67]. Another gene among the 39 genes is *Tudor-SN staphylococcal nuclease*, which is a stress granule protein-coding gene activated by oxygen, osmotic and temperature stress [70]. These results suggest that active flight causes molecular damage due to changes in cellular processes and leads to a stressful condition. Recovering from the damage is a slow process, and it is hence not unexpected that the signal of repair would be detected as high base-line expression of the relevant genes in fragmented populations consisting of frequently flying butterflies.

The single most distinct group of genes that were highly expressed at 20 hrs after the flight treatment and which were significantly more expressed in fragmented than continuous landscapes were antimicrobial peptide (AMP) genes. The expression of AMPs is regulated by *Toll* [71] and *NF-K*b [72] signalling pathways as well as by ecdysteroids [73],[74], which may lead to a connection between hexamerin and AMP expression (above). Given the up-regulation of AMP genes following active flight, and the finding that active flight enhances a general immune response (encapsulation rate) in the Glanville fritillary [75], we attribute the high expression of AMP genes in fragmented landscapes to flight-induced stress in frequently-flying butterflies. In *Drosophila*, the expression of AMPs has been linked to oxygen stress in hypoxia [76]. In low-oxygen selection lines, tolerant flies up-regulated genes in signal transduction pathways, e.g. *Notch* and *Toll/Imd* pathways [76], while experimental overexpression of one antimicrobial peptide (*Diptericin*) in a hyperoxic environment [77] caused altered gene expression in many of the same genes and functional categories that we observed to be differentially expressed between fragmented and continuous populations.

## Conclusion

Theoretical studies predict [13],[18] and empirical studies confirm [12],[17],[78] that butterflies in highly fragmented landscapes are especially dispersive, have high post-diapause larval growth rate (Fig. 1d,e; [19]) and have high rate of egg maturation and reproduction [44],[79]. Populations in fragmented landscapes thus have a “fast” life history, which is consistent with high expression of hexamerin genes and *Ace* in new populations and in fragmented landscapes. High flight metabolic rate allows high dispersal rate [58], but it may potentially involve the cost of hypoxia. We found that butterflies from new populations and from fragmented landscapes exhibit low expression of *Hif-1a* and high expression of AMP genes, which enhance their tolerance of hypoxia and thereby facilitate flight activity. We conclude that significant differences in gene expression between butterflies from different landscape types, measured under common garden conditions, highlight genetic adaptation to living in fragmented landscapes, where natural selection favors a fast life history.

## Supporting Information

Figure S1 **BAPS analysis of population clustering.** Top row shows the result of the primary analysis in which 174 individuals were divided into two groups, SA *versus* the three other populations. When SA was not included and the number of clusters was forced to be three (bottom row), ÖL formed one cluster and ÅL together with UP formed another cluster. One individual of ÖL (red) was assigned to a third cluster and two individuals, one from ÖL and another one from UP, appeared to have switched labels. These three outliers were removed from further analyses.
(TIF)

Figure S2 **The amount of RNA-seq data per individual.** Individuals are on the horizontal axis and the number of reads on the vertical axis. White, gray and color indicate the numbers of raw, trimmed and mapped reads, respectively. Yellow, green, blue and red denote the populations ÅL, ÖL, SA and UP, respectively.
(TIF)

Figure S3 **Within-population genetic diversity (see Materials and Methods).** The panel on the left shows the percentage of polymorphic SNPs out of 103,606 SNPs, the panel on the right shows the percentage of non-shared SNPs.
(TIF)

Figure S4 **Distribution of normalized read counts for 16,667 genes.** The read count for each gene is the average for 15 individuals from ÅL.
(TIF)

Table S1 **Sample size for the four populations shown in** **Fig. 1a** **.** The three outliers detected by the BAPS analysis (Fig. S3) are included in this table.
(DOCX)

Table S2 **Distribution of the number of RNA-seq reads among 174 individuals.** Column ‘rawCount’ gives the number of original reads, ‘trimCount’ the number of reads after trimming, and ‘mapCount’ the number reads mapped on genomic scaffolds. ‘map%’ was calculated relative to trimmed reads. Column ‘inGenes’ gives the number of reads mapped within annotated gene models, and ‘inGenes%’ was calculated relative to the number of mapped reads.
(DOCX)

Table S3 **The significantly enriched GO and KEGG enrichment categories based on Fisher's exact test.**
(XLSX)

Table S4 **The 1,841 genes differentially expressed between populations from fragmented landscapes vs. continuous landscapes.**
(XLSX)

Table S5 **Genes in Wnt, Notch and hedgehog signaling pathways.**
(XLSX)

Table S6 **The 233 genes in** **Fig. 4** **.**
(XLSX)

Table S7 **The 70 SNPs in 60 genes that showed significant genotype-expression association.**
(XLSX)

Table S8 **The 39 genes in** **Fig. 6** **.**
(XLSX)
