# Correction: Transcriptome Analysis Reveals Signature of Adaptation to Landscape Fragmentation

**DOI:** 10.1371/journal.pone.0104668

**Published:** 2014-07-31

**Authors:** The PLOS ONE Staff

Figure 3 is incorrect. The authors have provided a corrected version here.

**Figure 3:**
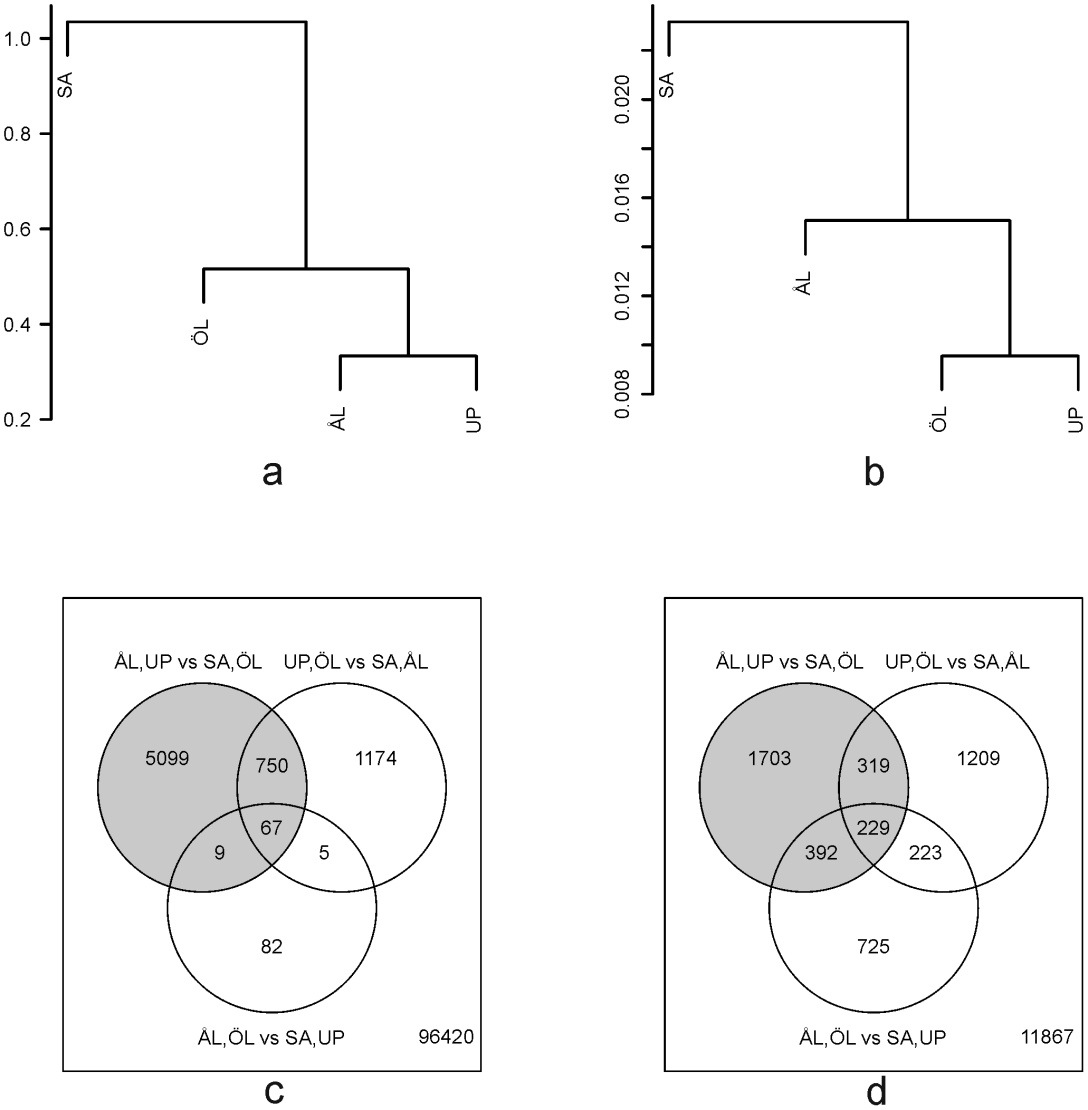
Clustering of the four study populations based on allele frequencies (a) and logarithmic gene-specific read counts (b). Distance was defined as one minus correlation. The lower panels show the Venn diagrams for the number of SNPs between pairs of populations with statistically significant (FDR<0.05) allele frequencies (c; Fisher's exact test) and for the number of statistically significant (FDR<0.05) differentially expressed genes (d; edgeR analysis). The pairs of populations representing the contrast between fragmented *versus* continuous landscapes are shown by gray shading.
