# Supplementary figures and images for: Transcriptome Analysis Reveals Signature of Adaptation to Landscape Fragmentation
Source: PLoS One. 2014 Jul 2;9(7):e101467. doi: 10.1371/journal.pone.0101467 (PMC4079591; doi:10.1371/journal.pone.0101467)

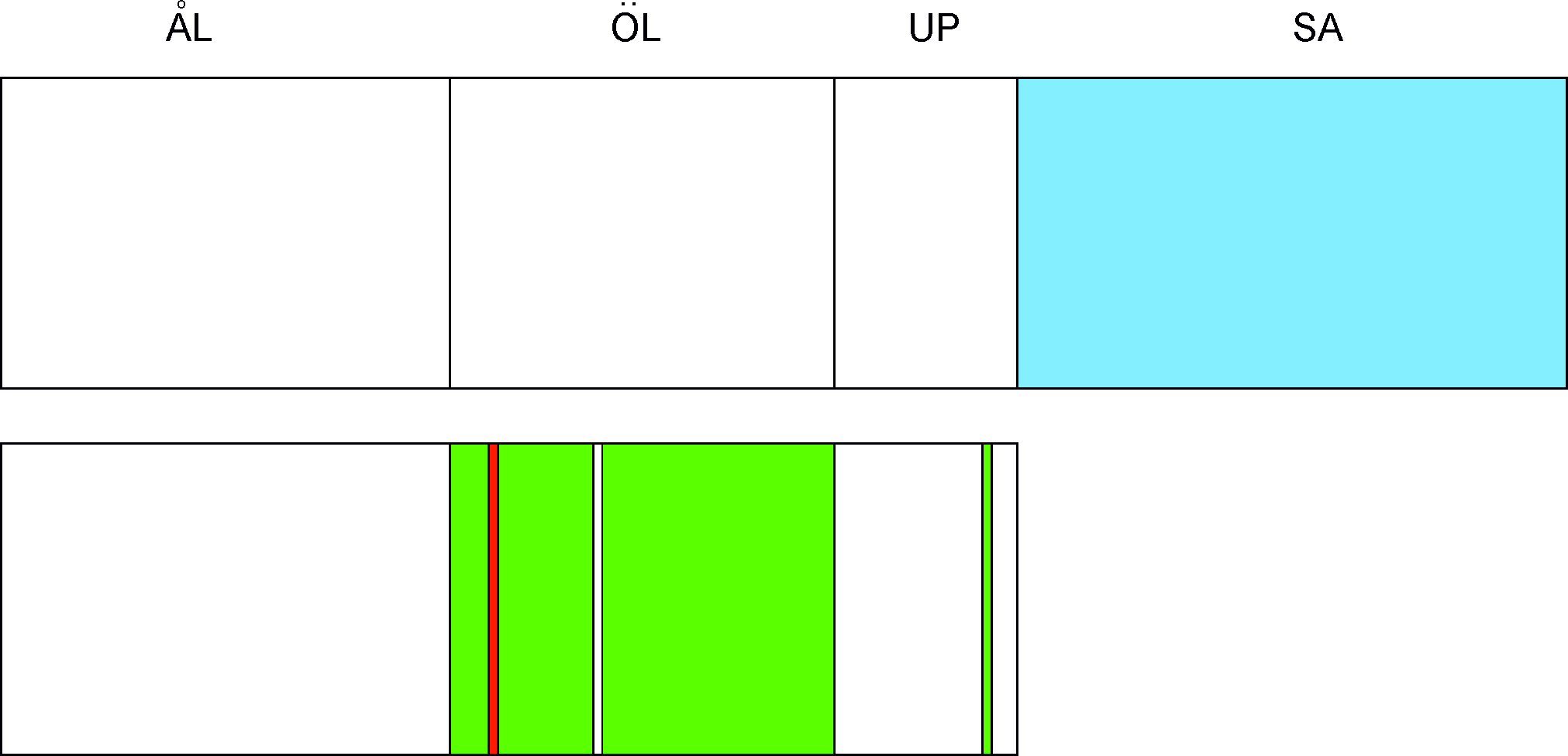

Supplement: Figure S1 — BAPS analysis of population clustering. Top row shows the result of the primary analysis in which 174 individuals were divided into two groups, SA versus the three other populations. When SA was not included and the number of clusters was forced to be three (bottom row), ÖL formed one cluster and ÅL together with UP formed another cluster. One individual of ÖL (red) was assigned to a third cluster and two individuals, one from ÖL and another one from UP, appeared to have switched labels. These three outliers were removed from further analyses. (TIF) [file pone.0101467.s001.tif]

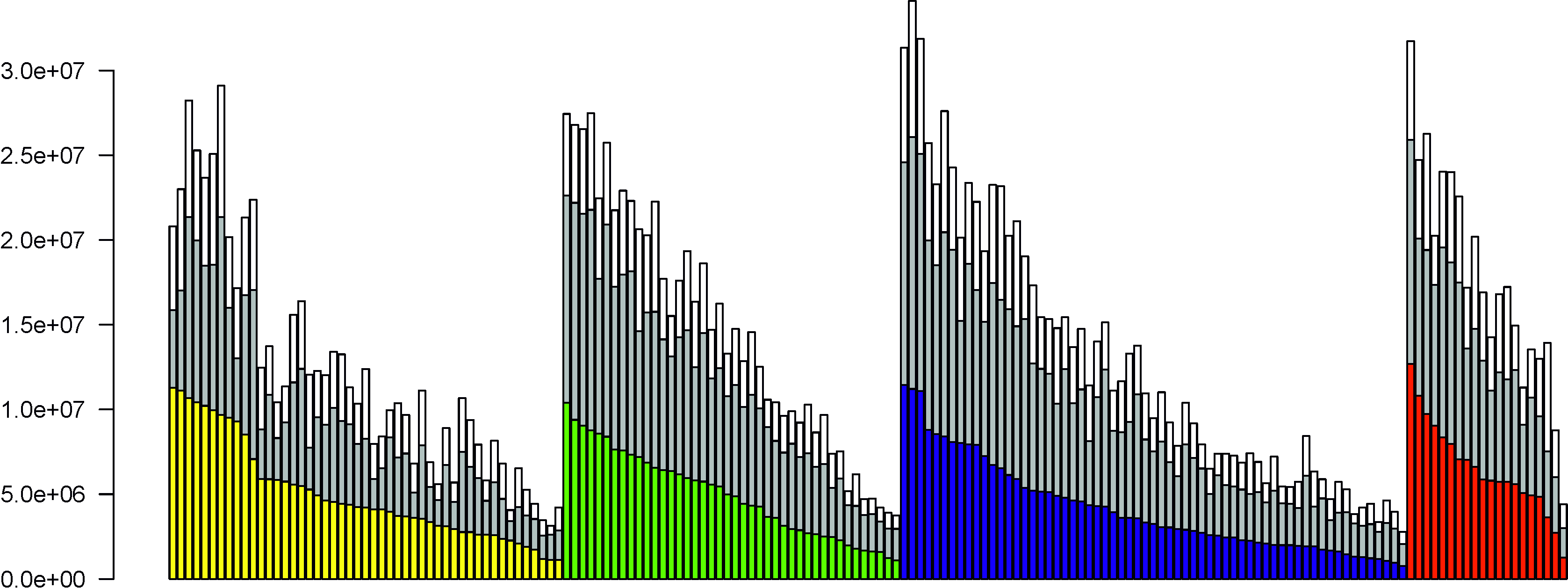

Supplement: Figure S2 — The amount of RNA-seq data per individual. Individuals are on the horizontal axis and the number of reads on the vertical axis. White, gray and color indicate the numbers of raw, trimmed and mapped reads, respectively. Yellow, green, blue and red denote the populations ÅL, ÖL, SA and UP, respectively. (TIF) [file pone.0101467.s002.tif]
