# Supplementary material for: Transcriptome Analysis Reveals Signature of Adaptation to Landscape Fragmentation
Source: PLoS One. 2014 Jul 2;9(7):e101467. doi: 10.1371/journal.pone.0101467 (PMC4079591; doi:10.1371/journal.pone.0101467)
Supplement: Table S1 — Sample size for the four populations shown in Fig. 1a . The three outliers detected by the BAPS analysis (Fig. S3) are included in this table. (DOCX) [file pone.0101467.s005.docx]

**Table S1. Sample size for the four populations shown in Fig. 1a.** The three outliers detected by the BAPS analysis (Fig. S1) are included in this table.

|  | females | males |
| --- | --- | --- |
| ÅL | 23 | 26 |
| ÖL | 15 | 27 |
| SA | 28 | 35 |
| UP | 11 | 9 |
