# Supplementary material for: Transcriptome Analysis Reveals Signature of Adaptation to Landscape Fragmentation
Source: PLoS One. 2014 Jul 2;9(7):e101467. doi: 10.1371/journal.pone.0101467 (PMC4079591; doi:10.1371/journal.pone.0101467)
Supplement: Table S2 — Distribution of the number of RNA-seq reads among 174 individuals. Column ‘rawCount’ gives the number of original reads, ‘trimCount’ the number of reads after trimming, and ‘mapCount’ the number reads mapped on genomic scaffolds. ‘map%’ was calculated relative to trimmed reads. Column ‘inGenes’ gives the number of reads mapped within annotated gene models, and ‘inGenes%’ was calculated relative to the number of mapped reads. (DOCX) [file pone.0101467.s006.docx]

**Table S2.** **Distribution of the number of RNA-seq reads among 174 individuals.** Column ‘rawCount’ gives the number of original reads, ‘trimCount’ the number of reads after trimming, and ‘mapCount’ the number reads mapped on genomic scaffolds. ‘map%’ was calculated relative to trimmed reads. Column ‘inGenes’ gives the number of reads mapped within annotated gene models, and ‘inGenes%’ was calculated relative to the number of mapped reads.

|  | rawCount | trimCount | trim% | mapCount | map% | inGenes | inGenes% |
| --- | --- | --- | --- | --- | --- | --- | --- |
| Min | 2774623 | 2071001 | 54.0 | 764302 | 32.0 | 442717 | 55.0 |
| 1stQu | 7388552 | 5468520 | 74.0 | 2588146 | 41.0 | 1514589 | 58.3 |
| Median | 12335934 | 9113574 | 77.0 | 4333682 | 45.0 | 2677838 | 60.0 |
| Mean | 13695253 | 10512133 | 76.4 | 4848724 | 46.1 | 2906692 | 59.8 |
| 3rdQu | 19931109 | 14869942 | 80.0 | 6550289 | 49.0 | 3997826 | 61.0 |
| Max | 34102489 | 26081050 | 86.0 | 12686182 | 71.0 | 7742394 | 67.0 |
